# Lipidomic and Metagenomic Profiling of Chinese Female Emerging Adults With Oily Scalp

**DOI:** 10.1111/jocd.70714

**Published:** 2026-02-05

**Authors:** Fang Yang, Baoyu Xiang, Dandan Xia, Yaoyao Wu, Xiaowei Chang, Peiwen Sun, Menghui Zhang, Yan Zhang

**Affiliations:** ^1^ Key Laboratory of Systems Biomedicine (Ministry of Education), Shanghai Center for Systems Biomedicine, State Key Laboratory of Microbial Metabolism, Joint International Research Laboratory of Metabolic & Developmental Sciences, School of Life Sciences and Biotechnology Shanghai Jiao Tong University Shanghai China; ^2^ PROYA Shanghai R&D Center Shanghai China

**Keywords:** *Lawsonella clevelandensis*, *Malassezia globosa*, *Malassezia restricta*, oily scalp, scalp surface lipid

## Abstract

**Background:**

Excessive sebum secretion leads to oily scalps, which can disturb microbial homeostasis and cause various scalp issues, such as sensitive scalp, dandruff, and seborrheic dermatitis.

**Aims:**

This study aimed to investigate the characteristics of scalp lipids and microbiota in a group of females with excessive sebum secretion using omics technology, and to identify important relationships between feature lipids and dominant functional microbes on oily scalp.

**Methods:**

Through comparison of three lipidomic sampling methods, we first selected absorbent paper (AP) as a cost‐effective and practical method for untargeted lipidomic profiling. Using this method, we then collected scalp surface lipids from 85 Chinese female emerging adults with varying degrees of excessive sebum and performed internal standard quantified lipidomic profiling using UPLC‐QE Plus‐MS equipped with LipidSearch software version 5.1. Simultaneously, we collected and analyzed scalp microorganisms using PE150 pair‐end metagenomic sequencing on the Illumina NovaSeq platform followed by taxonomic and functional annotation with bioinformatic tools and databases. Afterwards, multivariate statistical analysis and bioinformatics were used to identify feature lipids related to high sebum levels, discern the roles of dominant microbes involved in lipid metabolism, and explore potential correlations between feature lipids and dominant functional microbes of oily scalp.

**Results:**

After comparison of three lipidomic sampling materials, absorbent paper (AP) was selected to collect scalp surface lipids from 85 volunteers. A total of 13 lipid classes were annotated and the most abundant in ESI (+) mode was triacylglycerol (TG, 99.18%) whereas in ESI (−) mode were fatty acid (FA, 56.94%) and O‐acyl‐(gamma‐hydroxy) FA (OAHFA, 34.15%). We identified 27 TGs and 3 FAs as the major lipid molecules contributing to high sebum levels. Seventy percent of these TGs were unsaturated (33% monounsaturated, 26% diunsaturated, 11% triunsaturated), and 30% were saturated. Meanwhile, we found that although the dominant microorganisms, *Cutibacterium*, *Lawsonella*, *Malassezia*, and *Staphylococcus* were all involved in lipid metabolism on the scalp, only some of them were related to the degree of sebum level and also displayed species‐specific preferences for lipids. Among them, *Lawsonella clevelandensis* and *Malassezia globosa* were weakly negatively associated with both unsaturated and saturated TGs, while *Malassezia restricta* and *Cutibacterium granulosum* were only weakly negatively correlated with saturated TGs, and *Cutibacterium namnetense* was weakly positively correlated with FA (26:0).

**Conclusions:**

This study describes relevant lipid molecules contributing to higher sebum production, and reveals that *L. clevelandensis*, *M. restricta*, *M. globosa* , *C. namnetense*, and *C. granulosum* on the scalp are closely correlated with these lipids, showing species‐specific preference. These findings provide new insights into the interaction between key surface lipids and dominant functional microorganisms on oily scalps.

**Abbreviations**
3S: Sensitive Scalp Score
AP: absorbent paper
ASFS: adherent scalp flaking scale
Cer: ceramide
CerP: ceramide 1‐phosphates
DB: double bond
DG: diglyceride
DS: D‐squame
ESI: electrospray ionization
FA: fatty acid
GC–MS/MS: gas chromatography–mass spectrometry
GO: Gene Ontology
Hex1Cer: ceramide monosaccharides
HPLC‐MS/MS: high performance liquid chromatography‐mass spectrometry
MDA: mean decrease accuracy
MGDG: monogalactosyldiacylglycerol
OAHFA: O‐acyl‐(gamma‐hydroxy) fatty acid
PA: phosphatidic acid
PE: phosphatidylethanolamine
PG: phosphatidylglycerol
PI: phosphatidylinositol
RF: Random Forest
SFA: saturated fatty acid
ST: sebutape
TEWL: transepidermal water loss
TG: triglyceride
UFA: unsaturated fatty acid
UPLC‐QE Plus‐MS: ultra‐performance liquid chromatography & Q Exactive Plus mass spectrometer
VAS: Visual Analogue Scale
WE: wax monoesters

## 1 Introduction

With the influence of lifestyle and dietary changes, excessive sebum secretion on the scalp among young people in China has become increasingly prevalent. Moderate sebum secretion protects the scalp, and scalp surface lipids not only form a protective barrier but also serve as critical nutritional sources and signaling molecules for colonized microorganisms [1]. Scalp microbes degrade sebum to produce and uptake external fatty acids for survival, and their metabolites can further affect scalp health [2, 3]. Excessive sebum secretion by sebaceous glands creates an abundant nutrient supply for microbes, promoting their overproliferation and the production of irritating metabolites [4, 5]. This condition can also lead to increased transepidermal water loss (TEWL) and pH on the scalp, as well as elevated expression of inflammatory factors such as IL‐8, indicating impaired scalp barrier function [6]. These changes can stimulate the skin's immune system, resulting in scalp problems such as sensitive scalp, dandruff, seborrheic dermatitis, and hair loss [5, 7, 8].

Studies have found that sebum secretion is influenced by age and androgen levels, peaking during puberty [9]. Scalp diseases in young individuals, such as seborrheic dermatitis and androgenetic alopecia, have been the focus of attention for decades, particularly in males [10]. However, studies focusing on young female adults with excessive sebum secretion remain limited, hindering the development of effective products for oil control in this population. A previous study examining the scalp characteristics of women from six representative metropolises in China revealed that the relative amount of scalp sebum in Chinese women is three times higher than that in Korean women, peaking in their 20s and then significantly decreasing from their 40s [11]. In our preliminary market research, it was found that young Chinese women aged 18–25 tend to exhibit excessive sebum production. Although many show no obvious scalp diseases such as dandruff, seborrheic dermatitis, or hair loss, they frequently report uncomfortable sensations including scalp sensitivity and itching—clear indicators of deteriorating scalp health. This condition represents a critical early stage in the development of more severe scalp issues, and timely intervention is essential to mitigate the risk of progression. Although previous studies have explored the relationships between skin lipids and the skin microbiota across different body sites, skin types, and populations [12, 13], systematic investigations focusing on intra‐group variations in lipid levels and their associations with the microbiota in young females with oily scalps remain limited. Therefore, investigating both scalp surface lipid and microbial profiles in this youth group with oily scalps could provide useful insights for managing oily scalp, thus facilitating early intervention to prevent the deterioration of scalp problems.

Recent lipidomic‐based approaches, including high performance liquid chromatography–mass spectrometry (HPLC–MS/MS) and gas chromatography–mass spectrometry (GC–MS/MS), have been increasingly employed in skin and scalp studies. In the experimental workflow, sampling methods significantly impact lipid research outcomes. Currently, Sebutape (ST) and D‐squame (DS) are commonly used tape‐stripping sampling materials for skin or scalp surface lipids and stratum corneum lipids [5, 14, 15]. However, due to interference from scalp hair and background contamination from the sampling material, simpler and less polluted methods are needed.

In this study, we aimed to explore the lipid and microbial profiles of the scalp surface in young Chinese females with excessive sebum secretion. We first compared three sampling materials including absorbent paper (AP), ST, and DS for scalp surface lipid sampling and AP was selected for subsequent research. We then performed qualitative and quantitative lipidomic profiling of 85 Chinese female emerging adults with oily scalps using ultra‐performance liquid chromatography & Q Exactive Plus mass spectrometer (UPLC‐QE Plus‐MS). Simultaneously, we detected microbial profile using metagenomic sequencing technology. Finally, we identified lipids related to high sebum levels and explored the correlations between microbes and these lipids. Our findings provide novel insights into the relationship between lipids and microorganisms on oily scalps.

## 2 Materials and Methods

### 2.1 Volunteer Recruitment

**FIGURE 1:**
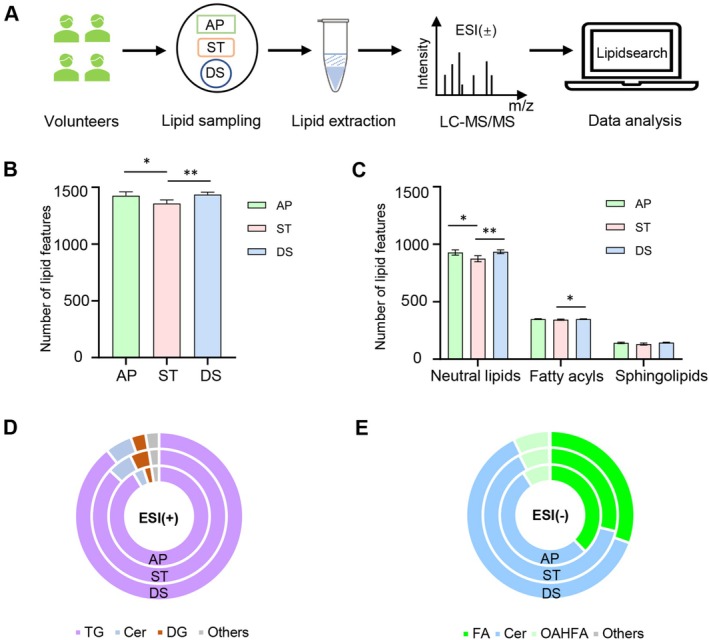
(A) Schematic representation of the comparative experiment on sampling materials for scalp surface lipid detection. AP, absorbent paper; DS, D‐squame; ST, sebutape. (B) The total numbers of lipid features. (C) The numbers of the top three major categories of lipid features. (D, E) The relative abundance of main lipids at class level in ESI (+) and ESI (−) mode, respectively. The *p*‐values were calculated by Mann–Whitney test. *p < 0.05, **p < 0.01.

**FIGURE 2:**
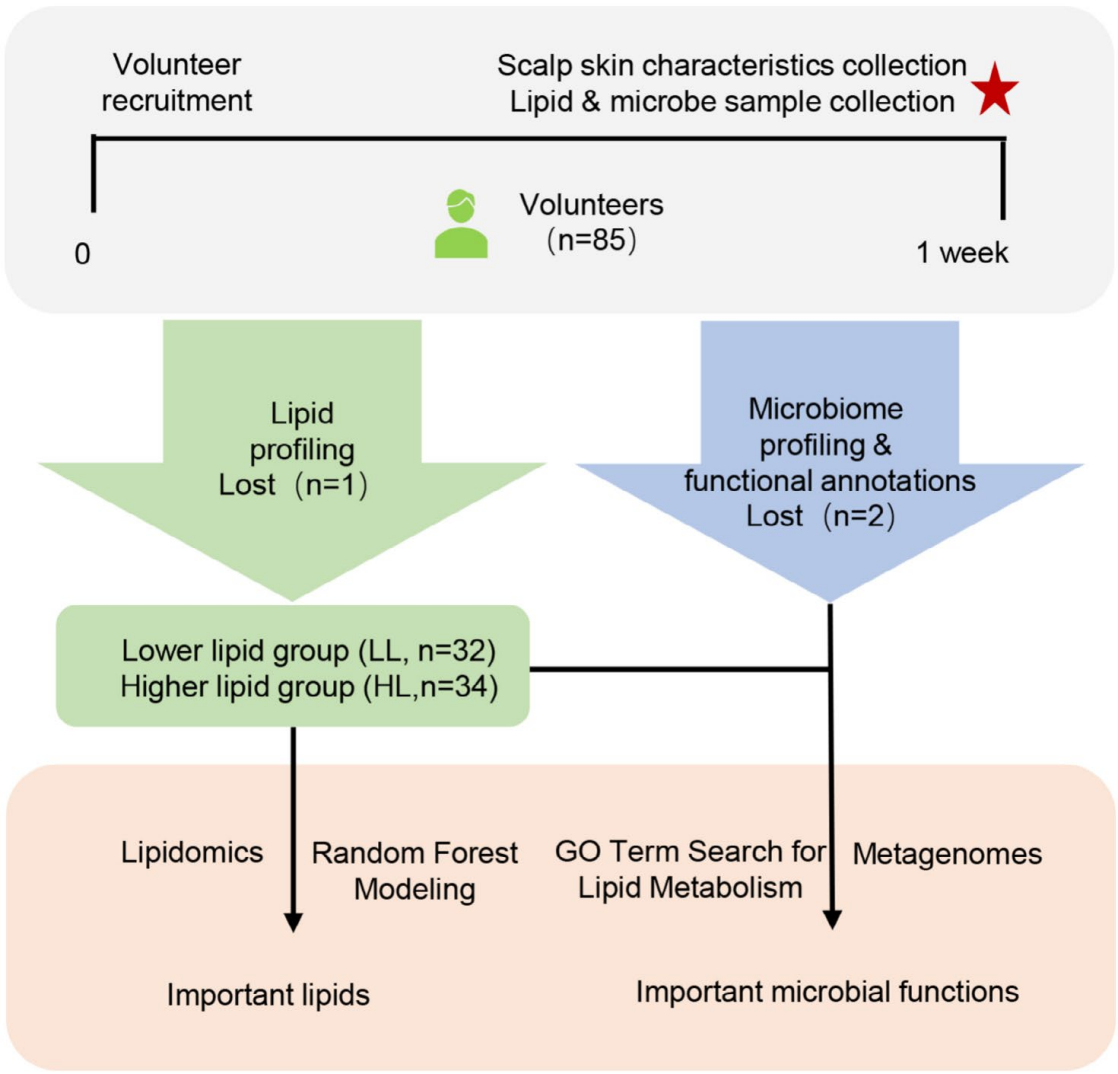
Workflow of the lipidomic and metagenomic profiling. The red asterisk represents the sampling time point.

A total of 219 female volunteers aged 18–25 were recruited and 85 of them were selected for study based on the criteria from one college located in Shanghai. Four of them were involved in the sampling material comparison experiment (Figure 1), and all volunteers participated in the lipidomic and metagenomic profiling experiment (Figure 2). Inclusion criteria encompassed female volunteers aged between 18 and 25 years, exhibiting no serious dandruff problems, and no other scalp or hair projects were currently being undertaken. The exclusion criteria were as follows: (1) recent clinical trial participation within 3 months; (2) taken of oral or topical hormones, antibiotics, contraceptives, antibacterial drugs, antihistamines, anti‐inflammatory drugs, or immunosuppressants within 3 months; (3) use of hair‐affecting products or dandruff products within 1 month; (4) hair transplantation, perming, dyeing, or bleaching within 1 month; (5) pregnancy or breastfeeding; (6) BMI > 30; (7) scalp conditions such as seborrheic dermatitis, psoriasis, allergic dermatitis, or eczema; and (8) any other health problems including mental or psychological diseases, or those with long‐term sleep or emotional control disorders.

The study was approved by the Scientific and Ethical Committee and performed in accordance with the approved guidelines and regulations. All volunteers signed informed consent forms explaining the procedure and purpose of the study.

### 2.2 Sample Collection

All volunteers had hair washed 48 h prior to sample collection and did not use any scalp or hair products afterward. On the day of sample collection, subjects stayed in a controlled environment with a relative humidity of 40%–60% and a temperature of 20°C–22°C.

#### 2.2.1 Physiological Parameter Measurements

The volunteers firstly completed Visual Analogue Scale (VAS) scoring [16, 17] for itchiness and Sensitive Scalp Score (3S) questionnaire [18]. Then the scalp of each volunteer was divided into eight divisions and the adherent scalp flaking scale (ASFS) level of each division was evaluated by a licensed dermatologist. Physiological measurements were performed on the vertex area of the scalp in the order of sebum, TEWL, hydration and pH. Sebum levels were measured using a Sebumeter SM 815 (Courage‐Khazaka, Germany). TEWL was assessed using the VapoMeter (Delfin Technologies, Finland). Scalp hydration was measured using the DermaLab Skinlab Combo (Cortex Technology, Denmark). Scalp pH was measured using a Skin‐pH‐Meter PH905 (Cortex Technology, Denmark). All equipment was operated by trained and experienced personnel.

#### 2.2.2 Scalp Lipid Sampling

In the sampling material comparison experiment, absorbent paper (AP, BKMAN, China), Sebutape (ST, CuDerm, USA), and D‐squame (DS, CuDerm, USA) were cut into the same size (2.0 × 0.6 cm) before sampling. Each sampling material was pressed on the sampling site for 15 s with a D‐Squame pressure instrument (D500, CuDerm, USA) providing uniform pressure. Two layers of each material were sampled at adjacent sites but not overlapping. Negative controls (materials without lipid sampling) were also prepared and placed into tubes. All samples were immediately extracted for lipids and analyzed using untargeted UPLC‐QE Plus‐MS (Thermo Fisher Scientific, USA).

In the lipidomic profiling of the 85 volunteers, scalp surface lipid samples were taken by AP according to the process mentioned above. Briefly, AP was pressed on the sampling site of the scalp vertex for 15 s with a D‐Squame pressure instrument. Two layers of AP were collected on the same sampling site of each subject and placed into a tube. Negative control APs were also prepared and placed into tubes. All samples were immediately stored on dry ice and then stored at −80°C until lipid extraction, after which they were detected by UPLC‐QE Plus‐MS with internal standard quantification.

#### 2.2.3 Scalp Microbial Sampling

Scalp microbiome samples were taken by rubbing a sterile saline‐soaked swab (catalog number PSNB20013, PersonalBio, China) on a 5 × 3 cm area of each subject's scalp for 20 strokes [19]. Negative control swabs were processed similarly with air exposure for the same duration. All samples were immediately stored on dry ice and then stored at −80°C until microbiome DNA extraction.

### 2.3 Lipid Extraction and Lipidomic Analysis

Lipid extraction was carried out in 2 mL polypropylene tubes using the Folch method with minor modifications [20]. ^13^C‐labeled palmitic acid (10 μg, MedChemExpress, USA) and ^13^C‐labeled Triolein (0.2 μg, MedChemExpress, USA) were added as internal standards to each sample. The final lipid extract was dissolved in dichloromethane/isopropyl alcohol/methanol (1/1/2, v/v/v) for UPLC‐QE Plus‐MS detection. Quality control samples were prepared by mixing 5 μL of each sample into a pooled sample and tested every 10 samples.

Samples were analyzed in both Electrospray Ionization positive (ESI(+)) and negative (ESI(−)) modes by UPLC‐QE Plus‐MS. An ACQUITY UPLC BEH C18 column (100 × 2.1 mm, 1.7 μm, Waters Corporation, USA) was used with 10 mM ammonium formate and 0.1% formic acid in acetonitrile/water (6/4, v/v) as mobile phase A, and 10 mM ammonium formate and 0.1% formic acid in isopropanol/acetonitrile (9/1, v/v) as mobile phase B. The ESI parameters were as follows: capillary temperature at 320°C, spray voltage at 3.2 kV (ESI(+)) and 3.0 kV (ESI(−)), S‐Lens radio frequency level at 50 V, scan range from 135 to 2000 Da, and full MS scan mode with a resolution of 70 000. Data‐dependent second‐order mass spectrometry scanning was conducted with a resolution of 17 500, automatic gain control set to 5e5, and an ion trap of 50 ms.

The raw data was processed by LipidSearch software version 5.1 (Thermo Fisher Scientific, USA). The normalization of lipidomic spectrum was performed using an in‐house program in the MatLab (R2023b, The MathWorks, USA) environment. Briefly, each spectrum was baseline corrected with those of the negative controls and then quantitatively adjusted with its own internal standard using peak area (^13^C‐labeled Triolein with [M+NH_4_]^+^ ion in positive mode; ^13^C‐labeled Palmitic acid with [M−H]^−^ ion in negative mode). Ions detected only in one individual and false positive results were excluded. The resulting lipid ion data were used for subsequent statistical analysis.

### 2.4 Microbial DNA Extraction, Metagenomic Sequencing, Compositional, and Functional Profiling

The microbial DNA extraction and metagenomic sequencing were performed at Personal Biotechnology (Shanghai, China). Total microbial genomic DNA of samples was extracted using the OMEGA MagBind Soil DNA Kit (M5635‐02) (Omega Bio‐Tek, USA) and sequenced using the Illumina NovaSeq platform (Illumina, USA) with the pair‐end PE150 strategy.

Low quality reads, adapters and short reads were first deleted by Fastp (version 0.23.2) with the following parameters: “‐l 50 ‐g ‐w 8 ‐W5 ‐5 ‐q 20 ‐u 30” [21]. Contaminant reads were removed using bowtie2 (version 2.5.1) by mapping the reads to the human genome database (GRCh38.p14) [22].

MetaPhlAn4 (version 4.0.6) was used to generate taxonomic profiling data with database version mpa_vOct22_CHOCOPhlAnSGB_202212 [23]. HUMAnN3 (version 3.7) was used to predict the gene families of each microorganism in each sample on the basis of the taxonomic profile from MetaPhlAn4, and their abundances were summed according to Gene Ontology (GO) annotations.

### 2.5 Statistical Analysis and Data Visualization

The Random Forest Modeling was performed with RStudio (Version: 2025.05.1+513, https://posit.co/download/rstudio‐desktop/). The correlation heatmap was plotted using https://www.bioinformatics.com.cn, an online platform for data analysis and visualization. Other statistical analyses and graphing were done by GraphPad Prism (version 8.02, USA). Two‐tailed Wilcoxon's tests for the non‐parametric data were applied for significance calculations. Network map analysis was performed with Cytoscape (version 3.10.1, USA).

## 3 Results

### 3.1 Selection of Sampling Materials for Scalp Surface Lipid

We conducted a comparative analysis of three sampling materials including AP, ST, and DS for untargeted lipidomic profiling analysis from four volunteers (Figure 1A). The total average number of lipid features identified by AP, ST, and DS sampling materials was 1426, 1357, and 1436, respectively, all belonging to the same 23 lipid classes and 40 subclasses (Figure 1B, Table S1). AP detected a higher feature number than ST (*p* < 0.05), and a comparable number to DS (*p* > 0.05). When comparing the total average number of the three major lipid categories, AP also showed similar results (Figure 1C). At the lipid class level, AP showed comparable relative abundance of dominant lipid classes to those of ST and DS (Figure 1D,E). In ESI (+) mode, the top three most frequently annotated lipid classes were triglyceride (TG), ceramide (Cer) and diglyceride (DG). In ESI (−) mode, the dominant classes were fatty acid (FA), Cer and O‐acyl‐(gamma‐hydroxy) fatty acid (OAHFA). Additionally, by comparing the total lipid content in negative control sampling materials, we found that AP had lower background detection than ST and DS (data not shown). These results suggest that AP achieved results comparable to those of ST and DS with lower background contamination. Considering the detection of scalp surface lipids, operational feasibility and background interference, we selected AP as the lipid sampling material for subsequent large‐scale population studies.

### 3.2 Lipidomic Profiling of the 85 Volunteers With Oily Scalps

The scalp physiological parameters (sebum, hydration, TEWL, pH, ASFS, VAS, and 3S) of the volunteers were summarized in Table 1. The average age of these subjects was 22 years old, and their average ASFS score was 0.32, indicating they had no apparent dandruff problem. Notably, these subjects had sebum ranging from 192 to 530 μg/cm^2^, with over 60% exhibiting sebum content > 350 μg/cm^2^, much higher than that on normal scalps [24]. In addition, the 3S of these subjects was 5.61 ± 3.67 (ranging from 0 to 15), with 99% exhibiting sensitive scalps to varying degrees. The VAS was 44.4 ± 25.27 (ranging from 0 to 90), with 90% experiencing itchy scalps to varying degrees. Based on observed scalp sensitivity and itchiness, it suggests a decline in scalp health, although the TEWL remains within the normal range (5.33–10.87) which indicates no significant skin barrier impairment. Furthermore, there was a positive correlation between the 3S and VAS scores (*r* = 0.55, *p* < 0.01).

**TABLE 1:** Sample information and physiological characteristics of recruited volunteers.

Age (year)	Sebum (μg/cm^2^)	Hydration (μS)	TEWL (g/hm^2^)	pH	ASFS	VAS	3S
22.07 (19–25)	376.5 (192–530)	39.65 (11.67–98.67)	8.15 (5.33–10.87)	5.19 (4.31–5.97)	0.32 (0–3)	44.4 (0–90)	5.61 (0–15)

**FIGURE 3:**
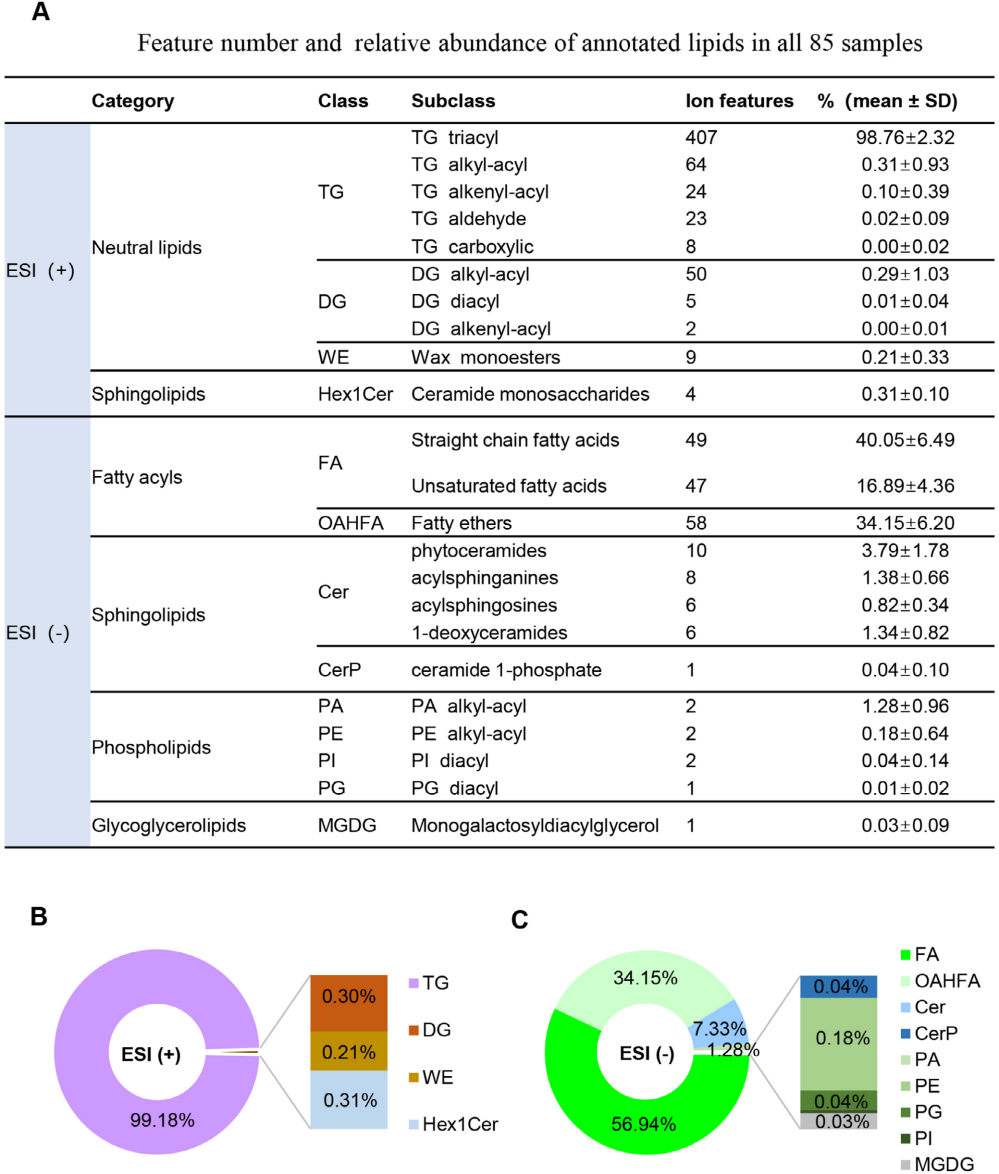
Scalp lipidomic profiling. (A) Numbers and relative abundance of the identified lipids. (B, C) Relative abundance of the lipids at class level in ESI (+) and ESI (−) mode, respectively.

A total of 2428 lipid features with [M+NH_4_]^+^ ions in ESI (+) mode and 258 lipid features with [M−H]^−^ ions in ESI (−) mode were initially detected. After data processing and manual verification, 596 lipid features in ESI (+) and 193 lipid features in ESI (−) were successfully annotated. These annotated lipids were classified into 5 categories, 13 lipid classes and 23 subclasses based on the LipidSearch database classification (Figure 3A). At the lipid class level, the annotated lipids in ESI (+) were TG (99.18%), Ceramide monosaccharides (Hex1Cer, 0.31%), DG (0.30%) and Wax monoesters (WE, 0.21%) (Figure 3B). In ESI (−), the top four lipid classes were FA (56.94%), OAHFA (34.15%), Cer (7.33%) and phosphatidic acid (PA, 1.28%) (Figure 3C). The relative abundances of other lipid classes, including phosphatidylethanolamine (PE), phosphatidylglycerol (PG), phosphatidylinositol (PI), monogalactosyldiacylglycerol (MGDG), and ceramide 1‐phosphates (CerP) were all less than 1%. These results indicate that TG and FA are the most abundant lipid components of the scalp surface, consistent with previous reports [25, 26].

### 3.3 Microbial Profiling of the 85 Volunteers With Oily Scalps

**FIGURE 4:**
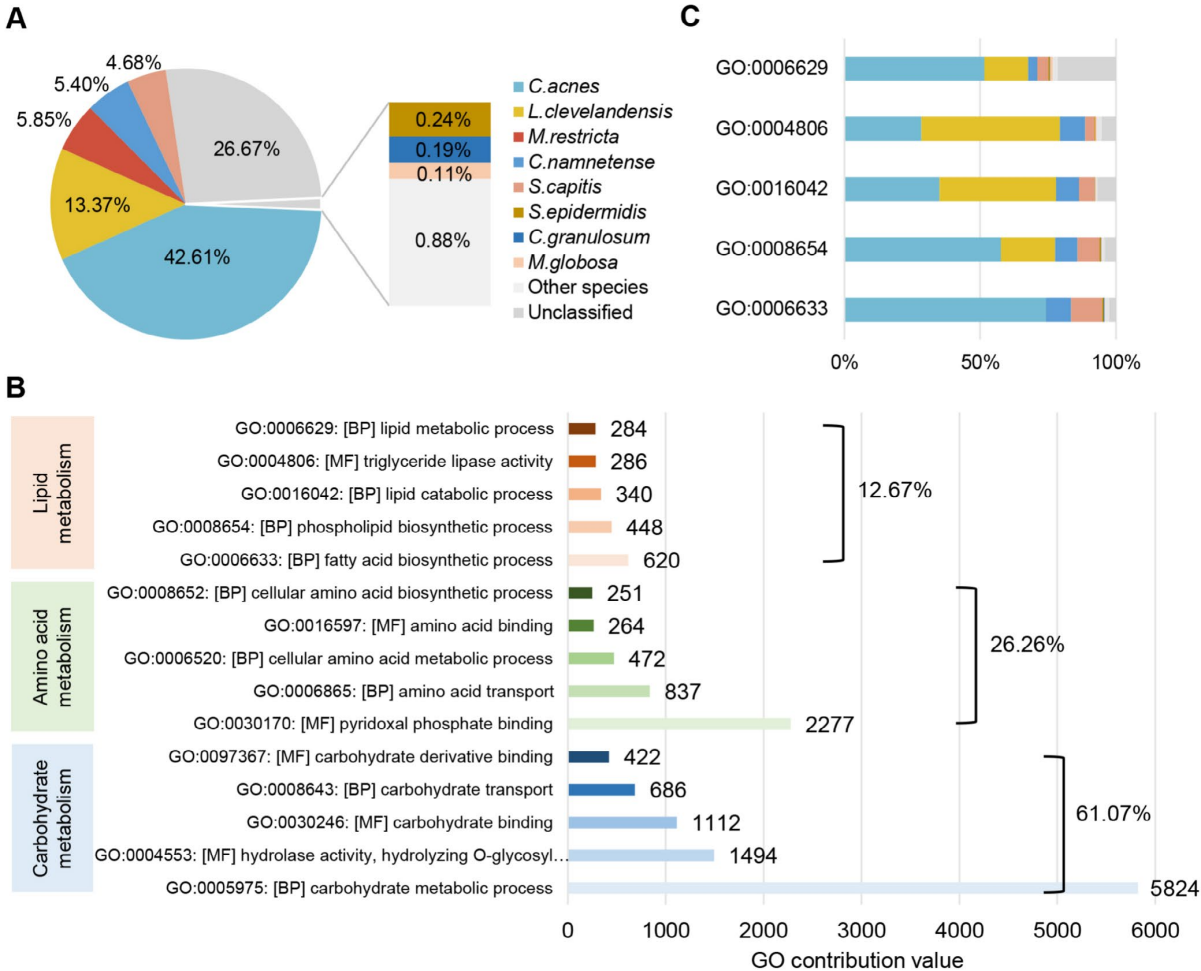
Scalp metagenomic profiling. (A) Relative abundance of the identified microorganisms. (B) The contribution value of the GO annotations related to lipid, amino acid, and carbohydrate metabolism. (C) Relative contributions of the microorganisms to lipid‐related GO annotation. The color for each microorganism was according to the Figure (A).

For the 85 volunteers, shotgun metagenomic sequencing yielded an average of 83.01 ± 14.14 million original reads per sample. After quality control and human contaminant removal, the average number of high‐quality reads was reduced to 35.74 ± 21.51 million per sample. A total of 258 microbial species were identified, with the eight dominant species being *Cutibacterium acnes* (42.61%), *Lawsonella clevelandensis* (13.37%), *Malassezia restricta* (5.85%), *Cutibacterium namnetense* (5.40%), *Staphylococcus capitis* (4.68%), *Staphylococcus epidermidis* (0.24%), *Cutibacterium granulosum* (0.19%), and *Malassezia globosa* (0.11%) (Figure 4A). *C. acnes* and *L. clevelandensis* accounted for more than 50% of the total microbial relative abundance. Unclassified microorganisms contributed 26.67% of the total, indicating a substantial proportion of uncharacterized microorganisms on the oily scalp.

With the metagenomic data, a total of 9216 GO terms were identified. To prioritize functionally relevant annotations, we sorted GO terms by their average contribution values in descending order and analyzed the top 300 GO terms related to three core metabolic processes. As shown in Figure 4B, carbohydrate metabolism‐related GO terms (61.07%) had the highest average contribution, followed by amino acid metabolism (26.26%) and lipid metabolism (12.67%). In the GO terms related to amino acid metabolism and carbohydrate metabolism, *C. acnes* was the microorganism with the greatest contribution, accounting for more than 50% (Figure S1). However, lipid metabolism showed a different profile. Among lipid metabolism‐related GO terms, the most prominent contributions were from the following five GO terms, fatty acid biosynthetic process (GO:0006633), phospholipid biosynthetic process (GO:0008654), lipid catabolic process (GO:0016042), triglyceride lipase activity (GO:0004806), and lipid metabolic process (GO:0006629). Different bacterial species exhibit variations in lipid utilization and metabolism. The synthesis of FA and phospholipids was mainly attributed by *C. acnes* (GO:0006633, GO:0008654), while lipid degradation and triglyceride lipase activity were mainly contributed by *L. clevelandensis* (GO:0016042, GO:0004806). *C. namnetense*, *S. capitis* and unclassified species also exhibited active contributions to lipid metabolism (Figure 4C).

### 3.4 The 30 Lipids Including 27 TGs and 3 FAs Related to Higher Lipid Amount

**FIGURE 5:**
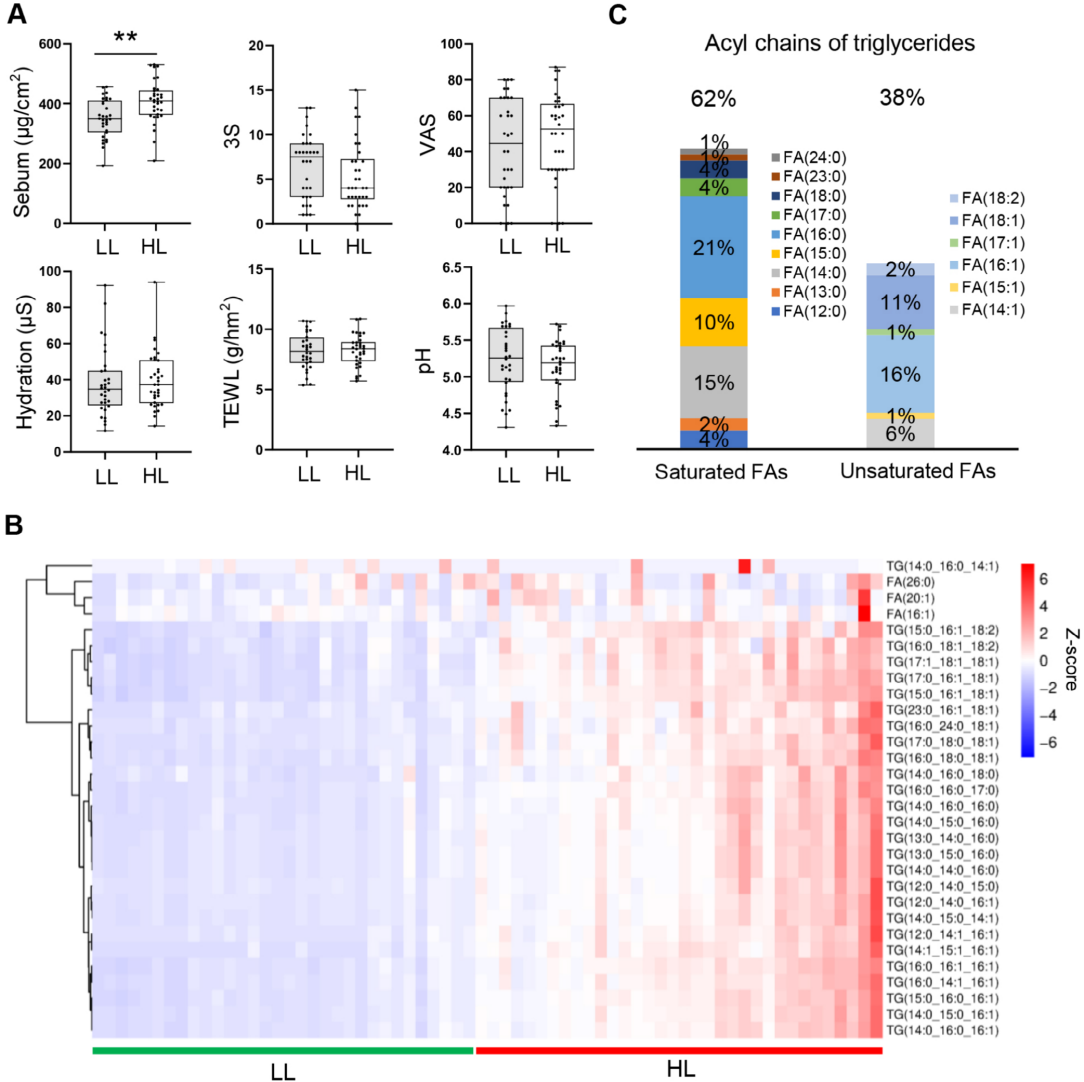
Identification of the oily‐scalp associated lipids. (A) Physiological indexes of the lower lipid (LL) and higher lipid (HL) group. (B) Heatmap of the content of oily‐scalp associated lipids in two groups. (C) Relative abundance of the acyl chains in the oily‐scalp associated TGs. **p < 0.01.

Both lipids and microorganisms displayed large variation among volunteers. These individual specific characteristics made it difficult to establish a linear relationship between lipids and scalp microorganisms. We therefore arranged the 85 volunteers in rank order according to the total lipid amount with respect to the internal standards. The total lipid amount ranged from 0.96 to 15.62, we selected the first 32 as the lower lipid group (LL, total lipid amount ranging from 0.96 to 2.92) and the last 34 as the higher lipid group (HL, total lipid amount ranging from 4.11 to 15.62) (Figure 2). The remaining 19 subjects within the intermediate range (3.04–3.96) were excluded to ensure clear stratification between the two groups. Consistent with the total lipid amount detected by UPLC‐QE PLUS‐MS, the HL group had significantly higher sebum (407.59 ± 70.56 μg/cm^2^) than the LL group (350.94 ± 64.14 μg/cm^2^, *p* < 0.01, Figure 5A), though sebum and total lipid amount did not significantly linear related.

To identify the lipids contributing to the higher total lipid amount, we conducted Random Forest (RF) analysis based on the detected lipids with sample coverage > 20%. The obtained RF model had good accuracy (97%) in classifying HL and LL samples. The top 30 lipids were defined as important lipids with the highest mean decrease accuracy (MDA) values in the RF analysis, where higher MDA reflects greater discriminatory importance. These lipids comprised 27 TGs and 3 FAs, all of which exhibited a positive correlation with total lipid amount (Figure 5B). The average abundance of the 30 lipids in the LL and HL groups was 34.30% and 43.45% of total lipid amount, respectively. Moreover, the 27 TGs grouped by their unsaturation degree‐double bond (DB) showed the following distribution: DB:1 (33%) > DB:0 (30%) > DB:2 (22%) > DB:3 (15%). Among the acyl chains of the 27 TGs, saturated fatty acids (SFAs) account for 62%, primarily consisting of C12‐C24 SFAs. The most abundant SFAs were FA (16:0) (21%), FA (14:0) (15%), and FA (15:0) (10%). Additionally, the remaining 38% were unsaturated fatty acids (UFAs), including monounsaturated and diunsaturated FAs with carbon chain lengths of C14–C18. The most abundant UFAs were FA (16:1) (16%) and FA (18:1) (11%) (Figure 5C). These TGs showed the same acyl chain composition as those detected on forehead [27]. Meanwhile, the three major free fatty acids were saturated fatty acid FA (26:0), monounsaturated fatty acid FA (16:1), and FA (20:1). Taken together, these results indicated that the 30 lipids might be used to reflect the scalp oily level.

### 3.5 Microbiota and Main Lipid Metabolism Functions Related to Higher Lipid Amount

**FIGURE 6:**
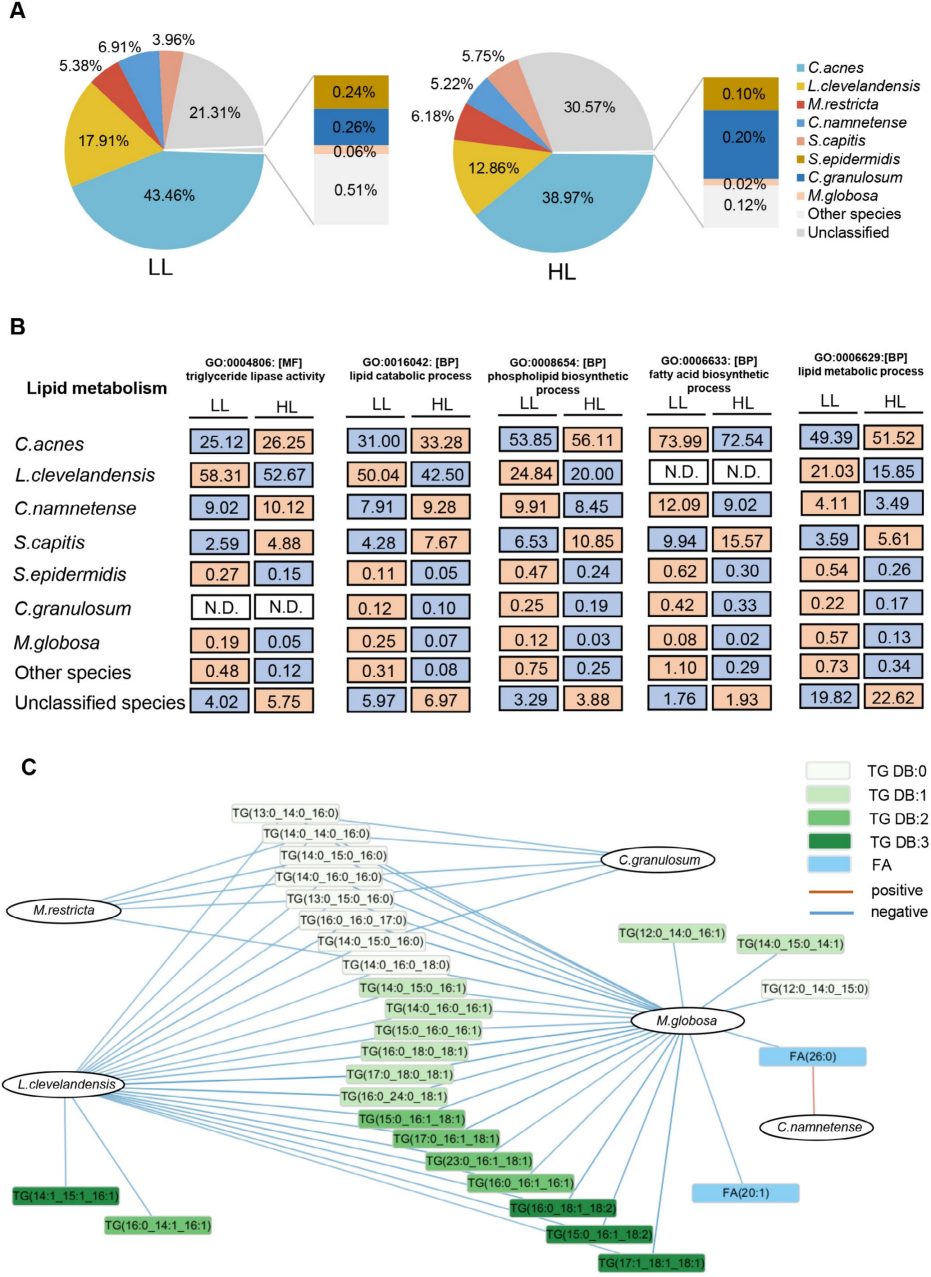
Comparison of the scalp microorganisms and microbial functions between the LL and HL groups. (A) Relative abundance of microorganisms in the two groups. (B) The relative contributions of each microorganism to the lipid‐related GO annotations. Orange: higher relative contributions; blue: lower relative contributions. N.D., not detected. (C) Correlation network of the microorganisms and oily‐scalp associated lipids. The correlations were calculated using Kendall's tau, and those *p* < 0.05 were shown.

Furthermore, the differences in microorganisms between the two groups were compared. The increased microbial species in the HL group included *M. restricta* (5.38% vs. 6.18%), *S. capitis* (3.96% vs. 5.75%), and unclassified microorganisms (21.31% vs. 30.57%) (Figure 6A). Notably, the unclassified microbes showed a 9.26% increase in relative abundance in the HL group. Conversely, *C. acnes*, *L. clevelandensis*, *C. namnetense*, *S. epidermidis* , *C. granulosum* , *M. globosa* , and other species were decreased in the HL group. This indicates that the abundance of microbial species has changed in a high lipid level.

We further compared microbial contributions to three core metabolic processes, particularly in five functional terms related to lipid metabolism (Figure 6B, Figure S2). The abundance of *C. acnes* had decreased in the HL group, but its contribution to triglyceride lipase activity and lipid catabolic process functions had not obviously changed. However, as the abundance of *L. clevelandensis* and *M. globosa* decreased, their contributions to these functions showed a downward trend. Notably, *S. capitis* and unclassified species both increased in abundance and functional contribution. Those results suggest that the abundance of various microorganisms and their inherent metabolic functions undergo different changes in the group with higher scalp surface lipids.

To further explore the associations of key microbiota and lipids, we performed Kendall correlation analysis between the microbes and the 30 important lipids (Figure 6C). We observed close correlations of lipids with *L. clevelandensis*, *C. namnetense*, *C. granulosum* , *M. restricta*, and *M. globosa* . Among them, *L. clevelandensis* and *M. globosa* exhibited weak negative correlations with most saturated and unsaturated TGs (*r* ranging from −0.25 to −0.15). *M. restricta* and *C. granulosum* showed only weak negative correlations with saturated TGs (*r* ranging from −0.17 to −0.15). We only observed a weak positive correlation between *C. namnetense* and FA (26:0) (*r* = 0.20). These results suggested that the interrelationship between microbes and lipids is species‐specific. In contrast, we did not observe significant associations between lipids and *C. acnes*, *S. epidermidis* , or *S. capitis* . Furthermore, we also analyzed the correlations within the 30 lipids. We found that all TG and FA molecules showed positive correlation within their respective classes, and there were also positive correlations between TGs and FAs (*r* ranging from 0.15 to 1, data not shown). Notably, eight saturated TGs showed the most correlations with microbes, including TG (12:0_14:0_15:0), TG (13:0_14:0_16:0), TG (13:0_15:0_16:0), TG (14:0_14:0_16:0), TG (14:0_15:0_16:0), TG (14:0_16:0_16:0), TG (14:0_16:0_18:0), and TG (16:0_16:0_17:0).

## 4 Discussion

The composition and quantity of scalp surface lipids are critical in maintaining the healthy status of the scalp and the homeostasis of colonized microbes. In this study, we found that absorbent paper is a convenient method for scalp surface lipid sampling, providing comparable lipidomic information to commonly used methods. Our lipidomic analysis of 85 Chinese female emerging adults with oily scalp indicated that 27 TGs and 3 FAs significantly contribute to the higher quantity of sebum lipids. The metagenomic analysis further identified that *L. clevelandensis*, *M. globosa* , *M. restricta*, *C. namnetense*, and *C. granulosum* are closely correlated with these 30 lipids.

We found that TG and FA were the most abundant scalp surface lipids, with TGs being the key players for higher oil scalp. This finding is consistent with previous studies that TGs and FAs were the most abundant lipids on the skin or scalp surface in both androgenetic alopecia and control groups [25, 26]. The sebum value is usually used to reflect the level of oiliness; however, it is still not clear which specific lipid molecules are related to the sebum levels. In our results, we found 30 lipids most related to sebum level by LC–MS analysis. Among these lipids, monounsaturated (DB:1) and saturated (DB:0) TGs were the most abundant in the 27 TGs of scalp surface lipids, differing from the TGs present on the forehead. On the forehead, monounsaturated (DB:1) and diunsaturated (DB:2) TGs were the most abundant among all detected TGs [28]. This observation indicates that saturated TGs account for a higher proportion on oily scalp than on forehead.

As the most abundant bacteria on skin or scalp, *C. acnes* and *S. capitis* have been reported to produce lipase to degrade lipids [2, 29], and we indeed found their contributions to lipid metabolism through bacterial source track in the functional analysis. However, we didn't observe a significant relationship between *C. acnes*/ *S. capitis* and lipids in the correlation analysis, suggesting that *C. acnes* and *S. capitis* are not related to the higher sebum level of oily scalps. Instead, we found that *L. clevelandensis* had wide and weak negative correlations with identified feature TG lipids. Notably, *L. clevelandensis* ranked second in abundance among the 85 subjects and contributed over 50% to GO terms related to lipid degradation, ranking first (Figure 4C). Through gene function analysis, we observed the important functional contribution of *L. clevelandensis* to lipid metabolism. Our results are also supported by recent studies that *L. clevelandensis* is more abundant in younger age groups [30] and it is a notable member of sebaceous sites such as the occiput of scalp [31]. *L. clevelandensis* was first separated from the *Cutibacterium* genus in 2016 but has been often overlooked [32, 33]. As a resident member of the healthy scalp microbiota in the Chinese population, the abundance of *L. clevelandensis* decreased in higher lipid group. This finding aligns with previous studies in Chinese population with healthy oily and dandruff oily scalps [7] and young South Korean atopic dermatitis patients [34]. However, a prior dandruff study reported elevated *L. clevelandensis* levels [19], highlighting microbiota variations based on the studied population and specific scalp conditions. Given its significant presence on scalp, the specific details of how *L. clevelandensis* utilizes lipids require in‐depth research.

The predominant *Malassezia* on the oily scalp were *M. restricta* and *M. globosa* , consistent with findings on healthy scalps [19, 26]. Although no functional annotations were mapped to *M. restricta* in this study, we observed a weak negative correlation between its abundance and the saturated TGs. *M. globosa* exhibited low abundance but showed a weak negative correlation with both saturated and unsaturated TGs, mirroring observations in healthy Japanese males [26]. *M. restricta* and *M. globosa* have been reported to secrete multiple lipases and uptake fatty acids degraded from sebum triglycerides as a nutrient source for survival [3]. *Malassezia* selectively utilize saturated FAs leading to a relative increase of unsaturated FAs such as oleic acid on scalp, which is believed to be the cause of dandruff [4, 35]. Consistently, our study found that *M. restricta* was negatively correlated with saturated TGs, suggesting that it overgrows on oily scalps where saturated TGs are elevated. This overgrowth may drive the accumulation of unsaturated FAs, raising the risk of dandruff formation. We observed an overall increase of *Malassezia* abundance in higher lipid group (data not shown), corroborating prior comparisons between healthy oily/dandruff scalps and non‐oily controls [7]. Notably, the abundance of *M. restricta* increased while that of *M. globosa* decreased in higher lipid group, a pattern also observed in Japanese males with androgenetic alopecia [26] and in multiracial populations suffering from dandruff and seborrheic dermatitis [36, 37]. Interestingly, a recent study on dandruff documented increased abundances of both *M. restricta* and *M. globosa* , highlighting potential discrepancies attributable to population differences or specific scalp conditions [19].

Two recent studies have reported correlations between skin lipids and microbiota [12, 13]. While these researches focus on skin surface and stratum corneum lipid components, our work investigates the scalp surface lipids using a non‐targeted approach. Although the research subjects and methodologies differ, both studies identified specific microorganisms associated with distinct lipids, such as oxylipins [12] and ceramides [13], also suggesting that microorganisms display species‐specific correlations with lipids. Collectively, these findings indicate that life stage, ethnicity, and body sites influence sebum production and microbial community composition, resulting in species‐specific microbial‐lipid interactions.

Existing research has shown that altering the scalp microbiome can lead to clinical improvements. A study on itchy scalp revealed that the colonization of non‐resident microbes from the environment is a potential key factor affecting scalp pruritus status. The severity of itch was alleviated when these low abundant and low subject coverage microbes were reduced or diminished [38]. Another study on dandruff showed that using shampoos containing traditional anti‐dandruff agents, such as Piroctone Olamine shampoo, can significantly change the scalp microbial composition and reduce harmful microorganisms like *M. restricta* to relieve dandruff problems [39]. A recent study on dandruff scalp further confirmed that shampoos effectively reduce dandruff and regulate microbial function, with the addition of anti‐dandruff ingredients enhancing these effects [19]. In this study, we identified important lipid molecules associated with excessive sebum production and established species‐specific correlations with several microorganisms, notably *L. clevelandensis*, *M. restricta* and *M. globosa* . For future oil‐control product development, we propose modulating microbial composition and biological functions through targeted interventions: reducing specific scalp lipids (e.g., saturated TGs), decreasing the abundance of *M. restricta*, *S. capitis* and unclassified microorganisms, while promoting the growth of *L. clevelandensis*. This approach aims to normalize microbial lipid metabolism and maintain scalp health. Sensitive skin or scalp is often linked to impaired skin barrier function [40]. This compromised barrier may facilitate irritant penetration and microbial colonization, potentially triggering dandruff or even seborrheic dermatitis through immune dysregulation [41]. Although no severe scalp conditions have been clinically observed in our study cohort, the presence of accompanying sensitivity symptoms suggests an elevated risk for developing dandruff and seborrheic dermatitis. If the composition and function of microorganisms as well as lipids are balanced, the common problems of sensitivity and itching that accompany high sebum levels may be alleviated and eliminated. This assumption awaits verification through large population experiments based on oil‐control products in the future.

While our research offers original insights, it is essential to acknowledge specific limitations. Since prior studies have reported the use of absorbent paper for facial lipid sampling [27], we compared the three sampling materials under identical conditions in four subjects. Our results suggest that absorbent paper meets experimental requirements for collecting scalp surface lipids, but it has limitations for sampling lipids from the scalp stratum corneum and would therefore be unsuitable for such applications. Given the limited number of subjects (*n* = 4) included in this comparative analysis, future studies are needed to validate the generalisability of AP using a larger sample size. Another limitation is that only females were involved, which might introduce biases due to hormonal levels, limiting the generalizability of the results. Therefore, studies involving larger and more diverse populations are necessary. Additionally, this study employed an internal comparative strategy by stratifying individuals with oily scalps according to total lipid content, aiming to minimize the inherent differences in sebaceous gland activity, barrier status and baseline microbial composition across different scalp types. Therefore, the present study did not incorporate a control group (e.g., volunteers with normal or dry scalps). The lipid and microbial profiles obtained here only reflect the status of the study population and do not represent the differences between oily scalps and normal or dry scalps.

In conclusion, through a systematic investigation of the lipid and microbial profiles of Chinese female emerging adults with oily scalp, we identified important lipid molecules that make significant contributions to high sebum levels and found their correlations with key resident microbes on the scalp. This study provides a research foundation for better understanding of oily scalp and the subsequent development of scalp care products.

## Author Contributions

Conceptualization: Y.Z., M.Z., and X.C.; Investigation: F.Y., D.X., and Y.W.; Project administration: Y.Z., X.C., and P.S.; Formal analysis: B.X., F.Y., and M.Z.; Writing draft: F.Y.; Writing and editing: M.Z. and Y.Z.

## Funding

This work was supported by Proya Cosmetics Co. Ltd.

## Ethics Statement

All the subjects were provided written informed consent willing to participate in the complete test process and had been explained the procedure and the purpose of the study. The study was reviewed and approved by the Scientific and Ethical Committee at the Shanghai Jiao Tong University with No. B20230321I.

## Conflicts of Interest

The authors declare no conflicts of interest.

## Supporting information


**Table S1:** Number of lipid features per lipid classes/subclasses with AP/ST/DS sampling.
**Figure S1:** Relative contributions of the microorganisms to the GO annotations related to (A) amino acid metabolism and (B) carbohydrate metabolism.
**Figure S2:** Comparison of the relative contributions of each microorganism to the GO annotations related to (A) amino acid metabolism and (B) carbohydrate metabolism between the LL and HL groups. Orange: higher relative contributions; blue: lower relative contributions. N.D., not detected.

## Data Availability

The sequencing data of this study are available from the corresponding author upon reasonable request.
