# Supplementary material for: Lipidomic and Metagenomic Profiling of Chinese Female Emerging Adults With Oily Scalp
Source: J Cosmet Dermatol. 2026 Feb 5;25(2):e70714. doi: 10.1111/jocd.70714 (PMC12875853; doi:10.1111/jocd.70714)
Supplement: Supplementary file 1 — Table S1: Number of lipid features per lipid classes/subclasses with AP/ST/DS sampling. Figure S1: Relative contributions of the microorganisms to the GO annotations related to (A) amino acid metabolism and (B) carbohydrate metabolism. Figure S2: Comparison of the relative contributions of each microorganism to the GO annotations related to (A) amino acid metabolism and (B) carbohydrate metabolism between the LL and HL groups. Orange: higher relative contributions; blue: lower relative contributions. N.D., not detected. [file JOCD-25-e70714-s001.docx]

**Lipidomic and metagenomic profiling of Chinese female emerging adults with oily scalp**

**Supplementary Material**

**Table Supplementary 1.** Number of lipid features per lipid classes/subclasses with AP/ST/DS sampling.

|  | **AP** | | | |  | **ST** | | | |  | **DS** | | | |
| --- | --- | --- | --- | --- | --- | --- | --- | --- | --- | --- | --- | --- | --- | --- |
| **Neutral lipids, sterols** | 1 | 2 | 3 | 4 |  | 1 | 2 | 3 | 4 |  | 1 | 2 | 3 | 4 |
| **Triglyceride（TG）** | **737** | **728** | **745** | **707** |  | **682** | **722** | **677** | **679** |  | **748** | **735** | **742** | **719** |
| TG-triacyl | 451 | 450 | 459 | 440 |  | 416 | 444 | 425 | 419 |  | 458 | 458 | 453 | 443 |
| TG-alkyl-acyl | 152 | 151 | 151 | 145 |  | 146 | 147 | 147 | 138 |  | 152 | 149 | 151 | 149 |
| TG-carboxylic | 64 | 58 | 62 | 54 |  | 59 | 62 | 43 | 55 |  | 67 | 59 | 62 | 59 |
| TG-aldehyde | 61 | 61 | 63 | 58 |  | 53 | 59 | 54 | 59 |  | 61 | 61 | 66 | 60 |
| TG-alkenyl-acyl | 9 | 8 | 10 | 10 |  | 8 | 10 | 8 | 8 |  | 10 | 8 | 10 | 8 |
| **Diglyceride（DG）** | **170** | **170** | **172** | **159** |  | **160** | **158** | **149** | **150** |  | **168** | **172** | **168** | **161** |
| DG-alkyl-acyl | 112 | 113 | 113 | 107 |  | 106 | 106 | 101 | 99 |  | 110 | 113 | 111 | 106 |
| DG-diacyl | 53 | 51 | 53 | 47 |  | 48 | 47 | 43 | 46 |  | 52 | 53 | 51 | 51 |
| DG-alkenyl-acyl | 5 | 6 | 6 | 5 |  | 6 | 5 | 5 | 5 |  | 6 | 6 | 6 | 4 |
| **Monoglyceride（MG）** | **11** | **10** | **11** | **10** |  | **8** | **11** | **7** | **12** |  | **10** | **10** | **12** | **9** |
| MG-monoalkyl | 6 | 5 | 6 | 5 |  | 3 | 5 | 4 | 6 |  | 6 | 5 | 6 | 5 |
| MG-monoacyl | 4 | 4 | 4 | 4 |  | 4 | 5 | 3 | 5 |  | 3 | 4 | 5 | 4 |
| MG-monoalkenyl | 1 | 1 | 1 | 1 |  | 1 | 1 | 0 | 1 |  | 1 | 1 | 1 | 0 |
| **Ch-cholesterol** | **18** | **18** | **18** | **18** |  | **18** | **18** | **18** | **17** |  | **18** | **18** | **18** | **18** |
| **AcHexSiE -acylHexSterolEsters** | **2** | **2** | **2** | **2** |  | **2** | **2** | **0** | **2** |  | **2** | **2** | **2** | **2** |
| **Zy-sterol** | **1** | **1** | **1** | **0** |  | **1** | **1** | **1** | **1** |  | **1** | **1** | **1** | **1** |
| **Fatty acyls** |  |  |  |  |  |  |  |  |  |  |  |  |  |  |
| **Fatty acid（FA）** | **44** | **45** | **44** | **45** |  | **41** | **42** | **32** | **44** |  | **44** | **44** | **44** | **43** |
| FA-Straight chain fatty acids | 25 | 24 | 25 | 24 |  | 22 | 22 | 13 | 25 |  | 25 | 23 | 25 | 22 |
| FA-Unsaturated fatty acids | 19 | 21 | 19 | 21 |  | 19 | 20 | 19 | 19 |  | 19 | 21 | 19 | 21 |
| **O-Acyl-(gamma-hydroxy) FA**  **（OAHFA）** | **17** | **17** | **17** | **17** |  | **17** | **13** | **11** | **16** |  | **17** | **17** | **17** | **17** |
| **Primary amides（PFAA）** | **27** | **26** | **30** | **24** |  | **28** | **29** | **26** | **27** |  | **28** | **29** | **30** | **27** |
| **N-Acylethanolamine（AEA）** | **21** | **21** | **22** | **16** |  | **20** | **21** | **19** | **20** |  | **21** | **22** | **22** | **21** |
| **Acyl carnitines（AcCa）** | **17** | **16** | **17** | **15** |  | **13** | **12** | **13** | **16** |  | **15** | **17** | **17** | **16** |
| **Coenzyme Q（Co）** | **1** | **1** | **1** | **1** |  | **1** | **1** | **1** | **1** |  | **1** | **1** | **1** | **1** |
| **Wax monoesters（WE）** | **15** | **17** | **17** | **15** |  | **15** | **15** | **17** | **16** |  | **17** | **16** | **17** | **16** |
| **Sphingolipids** |  |  |  |  |  |  |  |  |  |  |  |  |  |  |
| **Ceramide（Cer）** | **331** | **330** | **331** | **325** |  | **321** | **329** | **329** | **322** |  | **330** | **332** | **331** | **327** |
| Cer-acylsphingosines | 144 | 145 | 144 | 142 |  | 141 | 145 | 144 | 141 |  | 145 | 145 | 145 | 145 |
| Cer-acylceramides | 109 | 109 | 109 | 109 |  | 104 | 106 | 109 | 107 |  | 109 | 109 | 108 | 108 |
| Cer-phytoceramides | 55 | 55 | 55 | 55 |  | 54 | 55 | 53 | 52 |  | 54 | 55 | 55 | 53 |
| Cer-acylsphinganines | 19 | 17 | 19 | 15 |  | 18 | 19 | 19 | 18 |  | 18 | 19 | 19 | 17 |
| Cer-1-deoxyceramides | 4 | 4 | 4 | 4 |  | 4 | 4 | 4 | 4 |  | 4 | 4 | 4 | 4 |
| **CerP-dihydroceramide 1-phosphates** | **1** | **1** | **1** | **1** |  | **1** | **1** | **0** | **1** |  | **1** | **0** | **1** | **0** |
| **Hexosyl ceramide（Hex1Cer）** | **9** | **9** | **9** | **9** |  | **7** | **9** | **8** | **9** |  | **9** | **9** | **9** | **9** |
| Hex1Cer-Ceramide monosaccharides | 8 | 8 | 8 | 8 |  | 6 | 8 | 8 | 8 |  | 8 | 8 | 8 | 8 |
| Hex1Cer-t-Ceramide monosaccharides | 1 | 1 | 1 | 1 |  | 1 | 1 | 0 | 1 |  | 1 | 1 | 1 | 1 |
| **Sphingosine bases（SPH）** | **10** | **9** | **9** | **8** |  | **9** | **9** | **10** | **10** |  | **9** | **9** | **9** | **9** |
| SPH-Sphinganine | 1 | 1 | 0 | 1 |  | 0 | 0 | 1 | 1 |  | 0 | 1 | 1 | 0 |
| SPH-Sphingosine | 3 | 3 | 3 | 3 |  | 3 | 3 | 3 | 3 |  | 3 | 3 | 3 | 3 |
| SPH-deoxySphingosine | 4 | 3 | 4 | 2 |  | 4 | 4 | 4 | 4 |  | 4 | 3 | 3 | 4 |
| SPH-phyto-SPH | 2 | 2 | 2 | 2 |  | 2 | 2 | 2 | 2 |  | 2 | 2 | 2 | 2 |
| **Phospholipids** |  |  |  |  |  |  |  |  |  |  |  |  |  |  |
| **Phosphatidylethanolamine（PE）** | **2** | **2** | **2** | **2** |  | **1** | **2** | **2** | **2** |  | **2** | **2** | **2** | **2** |
| **Phosphatidylglycerol（PG）** | **1** | **1** | **1** | **1** |  | **0** | **1** | **1** | **1** |  | **1** | **1** | **1** | **1** |
| **Phosphatidylinositol（PI）** | **0** | **1** | **1** | **0** |  | **0** | **1** | **0** | **1** |  | **0** | **1** | **1** | **1** |
| **Phosphatidylcholine（PC）** | **1** | **1** | **1** | **0** |  | **1** | **1** | **1** | **1** |  | **1** | **1** | **1** | **1** |
| **Lyso phosphatidylinositol（LPI）** | **1** | **0** | **1** | **0** |  | **1** | **1** | **1** | **1** |  | **1** | **1** | **1** | **1** |
| **Glycoglycerolipids** |  |  |  |  |  |  |  |  |  |  |  |  |  |  |
| **MGDG-Monogalactosyldiacylglycerol** | **3** | **3** | **3** | **3** |  | **3** | **2** | **2** | **3** |  | **3** | **2** | **3** | **3** |
| **Total** | **1440** | **1429** | **1456** | **1378** |  | **1350** | **1401** | **1325** | **1352** |  | **1447** | **1442** | **1450** | **1405** |


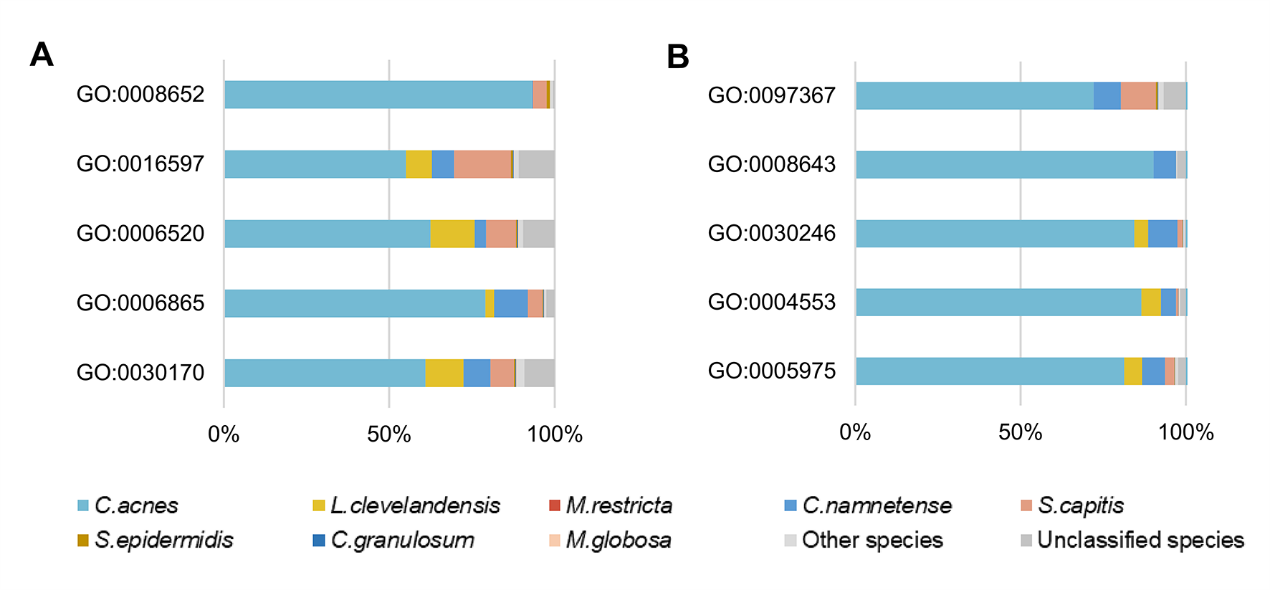


**Figure Supplementary 1.** Relative contributions of the microorganisms to the GO

annotations related to (A) amino acid metabolism and (B) carbohydrate metabolism.


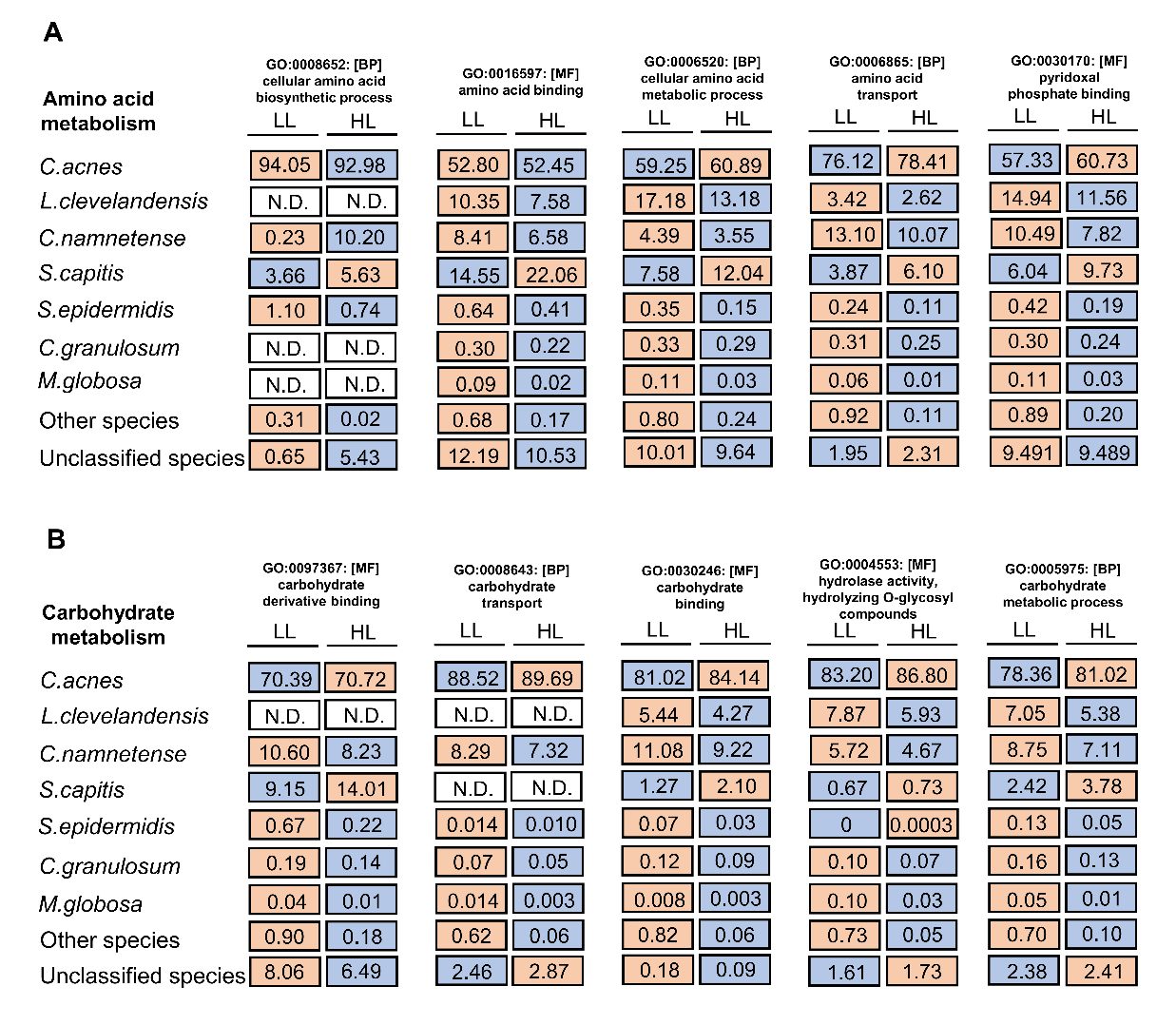


**Figure Supplementary 2.** Comparison of the relative contributions of each microorganism to the GO annotations related to (A) amino acid metabolism and (B) carbohydrate metabolism between the LL and HL groups. Orange: higher relative contributions; Blue: lower relative contributions. N.D.: Not Detected.
